# Metabolomics-based identification and validation of the creatine precursor guanidinoacetic acid for frailty in older adults

**DOI:** 10.1093/gerona/glaf127

**Published:** 2025-06-11

**Authors:** Yin Yuan, Xiaoming Huang, Siyang Lin, Wenwen Lin, Feng Huang, Pengli Zhu

**Affiliations:** Shengli Clinical Medical College, Fujian Medical University, Fuzhou, China; Fuzhou University Affiliated Provincial Hospital, Fuzhou, China; Shengli Clinical Medical College, Fujian Medical University, Fuzhou, China; Shengli Clinical Medical College, Fujian Medical University, Fuzhou, China; Fuzhou University Affiliated Provincial Hospital, Fuzhou, China; Shengli Clinical Medical College, Fujian Medical University, Fuzhou, China; Fuzhou University Affiliated Provincial Hospital, Fuzhou, China; Fuzhou University Affiliated Provincial Hospital, Fuzhou, China; Fujian Provincial Key Laboratory of Geriatrics, Fuzhou, China; Shengli Clinical Medical College, Fujian Medical University, Fuzhou, China; Fuzhou University Affiliated Provincial Hospital, Fuzhou, China

**Keywords:** biomarkers, frail older adults, guanidinoacetate

## Abstract

**Background:**

Subtle biological changes related to frailty may be undetected by standard clinical methods, and reliable biomarkers for frailty are still under investigation. This study was conducted to profile plasma metabolite patterns associated with frailty and validate the most significant metabolite for identifying and predicting frailty in cross-sectional and longitudinal analyses.

**Methods:**

The “Fujian Prospective Aging Cohort” (ChiCTR 2000032949) enrolled 2,265 community-dwelling individuals aged 60 and above in 2020. Plasma metabolites were analyzed using ultra-performance liquid chromatography-tandem mass spectrometry (UPLC-MS/MS). Frailty was assessed using Fried’s phenotype and the Frailty Index.

**Results:**

Widely targeted metabolomic analysis identified 889 metabolites. GAA was identified as the top frailty-associated candidate by ROC analysis and validated in a large cross-sectional cohort (AUC = 0.670). This cohort (N = 1,972) confirmed that subjects with lower GAA levels had a higher prevalence of frailty (*P* < .001). Multinomial logistic regression showed that higher GAA levels were significantly associated with lower odds of prefrailty and frailty; the ORs were 0.46 (95% CI: 0.32-0.66), and 0.15 (95% CI: 0.07-0.33) in the highest quartile, both *P* < .001). Over a 3-year follow-up period, a group-based trajectory model identified three Frailty Index trajectories: low-elevated (59.6%), moderate-elevated (34.1%), and high-elevated (6.3%). Subjects in the highest GAA quartile had a 36% and 66% lower likelihood of following moderate-elevated and high-elevated Frailty Index trajectories (*P* = .016 and *P* = .022).

**Conclusions:**

This study identifies GAA as a potential metabolic biomarker for frailty. Higher GAA levels are associated with lower frailty odds and provide predictive value for a lower likelihood of frailty progression.

## Introduction

Frailty, characterized by increasing vulnerability to adverse health outcomes, along with a reduced ability to maintain physiological balance, is increasingly recognized as a significant health challenge.^1^ As a potentially reversible condition, frailty underscores the need for early biomarkers to predict and prevent its onset effectively. Due to the heterogeneity of frail phenotype among individuals, various clinical tools have certain limitations in the screening and diagnosis for frailty.^2^ Many subtle biological changes associated with frailty may not be detectable using standard clinical methods, highlighting the need for innovative biomarkers for early detection. The molecular mechanisms of frailty are intricate, including inflammation, vascular endothelial cell damage, endothelial cell aging, oxidative stress, and other potential aspects.^3^ This complexity necessitates advanced diagnostic tools to identify specific biomarkers that can improve diagnosis and management. To date, reliable biological markers for frailty are still under investigation and require further validation.

Metabolomics, which analyzes the final products of genetic, transcriptomic, and proteomic activity, offers a promising approach to identifying frailty biomarkers. It connects inherent metabolic variations with frailty characteristics and functional decline.^4^^,^^5^ Previous studies have proposed various metabolites, such as amino acid metabolite profiles, acylcarnitines, and lipid derivatives (including fatty acids and glycerophospholipids), as potential indicators of frailty.^6–10^ However, no definitive conclusions have been reached. More importantly, it remains unclear how these candidate markers align with current frailty intervention strategies.

To address this knowledge gap, we undertook a 3-phase study: (1) identifying metabolic features associated with frailty in a community-dwelling older population using widely targeted metabolomic analysis; (2) validating the most significant metabolic candidate in a larger cohort by targeted metabolomic analysis; and (3) assessing its value for frailty identification and prediction in a longitudinal study. Our goal is to provide evidence for more accurate diagnosis and effective intervention strategies for frailty.

## Method

### Study design and population

The present study relies on “Fujian prospective aging cohort,” an ongoing prospective cohort aimed at assessing health condition based on comprehensive geriatric assessment in the non-hospitalized older population (ChiCTR 2000032949), as previously described.^11^^,^^12^ In May 2020, 2,265 individuals aged 60 years and above from Wenquan Community, Fuzhou City, were enrolled. Subjects with less than 6 months of life expectancy due to advanced malignancy or critical medical conditions and those unable to complete the questionnaire investigation and physical examination were excluded.

**Figure 1.**
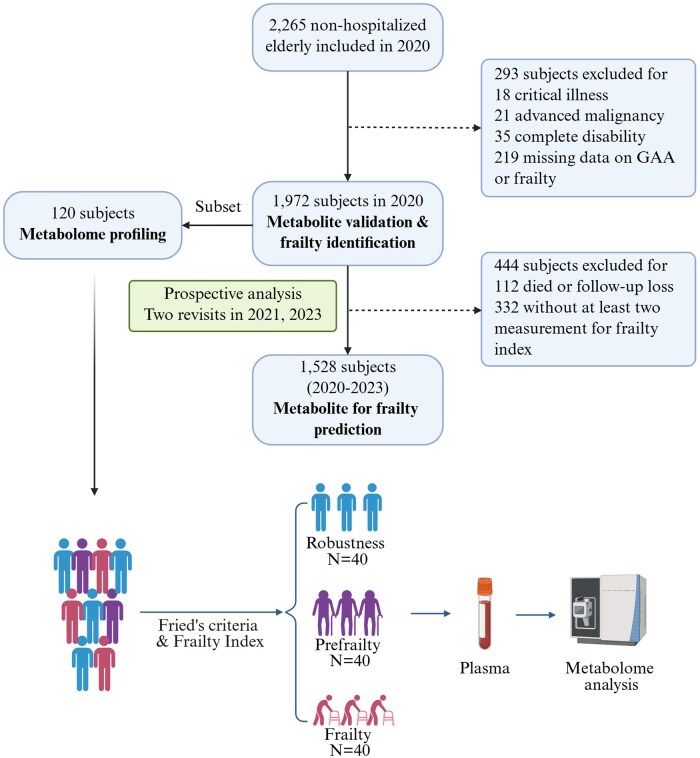
A schematic illustration of the study.

Figure 1 illustrates the flowchart of this study. Three datasets from the one cohort were utilized for the identification, validation, and evaluation of the predictive value of the selected metabolite associated with frailty. The discovery set, derived from the 2020 cohort wave, was used for widely targeted metabolomic analysis to identify plasma metabolites associated with frailty. To enhance credibility, frailty status was assessed using two well-established methods, Fried’s phenotype and the Frailty Index, both of which have been independently validated as strong predictors of adverse health outcomes.^13–15^ Participants were classified into three groups: frail, prefrail, and robust, based on their scores. Specifically, individuals were categorized as frail if they simultaneously met two criteria: having three or more components of Fried’s phenotype and a Frailty Index > 0.25. Participants were classified as prefrail if they met both of the following criteria: one or two components of Fried’s phenotype and a Frailty Index between 0.12 and 0.25. Those classified as robust simultaneously met two criteria: having no components of Fried’s phenotype and a Frailty Index < 0.12. The groups were matched for age (±3 years), gender, and body mass index (BMI) levels by propensity score matching strategy, with further inspection and adjustment to ensure balance across groups. The matching process yielded 40 frail, 40 prefrail, and 40 robust individuals, resulting in a total sample size of 120, consistent with the sample sizes reported in prior frailty-related metabolomic research.^5^^,^^6^ The first validation set (n = 1,972) consisted of cross-sectional data from the same cohort, excluding individuals without overnight fasting plasma samples and complete frailty-related data. To assess the predictive value of the metabolite for frailty progression over time at a prospective level, we conducted a longitudinal analysis using follow-up data from the second and third visits in July 2021 and September 2023. A total of 1,528 subjects with complete baseline data and at least one Frailty Index re-evaluation were included. This study received approval from the Ethics Committee of Fujian Provincial Hospital (No. K2020-05-008), in compliance with the Declaration of Helsinki. Written informed consent was obtained from all participants.

### Measurements

#### Covariates

Participants underwent a comprehensive geriatric assessment, during which various covariates were collected via questionnaires. These included demographic information, lifestyle factors such as exercise habits and protein intake frequency, and clinical indicators such as physician-diagnosed conditions and regular medications. Grip strength was measured using a hydraulic dynamometer (Jamar Dynamometer, Anaheim, CA), while gait speed was assessed with the 4-Metre Walk Test. Balance capacity was evaluated using the Timed Up and Go (TUG) test. Cognitive function was assessed using the Mini-Cog scale, with a score of ≤ 2 indicating cognitive impairment. Nutritional status was evaluated using the Mini Nutritional Assessment-Short Form (MNA-SF), with a score below 12 indicating a risk of malnutrition. Additional physical data collected included height, weight, and office blood pressure. Morning blood samples were taken after an overnight fast, and biochemical parameters such as creatinine, uric acid, homocysteine, blood lipids, fasting plasma glucose (FPG), and hemoglobin A1c (HbA1c) were measured.

### Definitions

Fried’s phenotype criteria include self-reported exhaustion, unintentional weight loss, slow walking speed (defined as the slowest 20% based on a 15-foot walking time, adjusted for gender and height), weakness (the lowest 20% of grip strength, stratified by gender and BMI quartiles), and low physical activity levels.^13^ The Frailty Index was constructed using 40 items based on Rockwood’s principles,^14^ as detailed in Table S1. Mean arterial pressure (MAP) was calculated using the formula: diastolic blood pressure plus one-third of the pulse pressure difference.

### Widely targeted and targeted metabolomic analysis

Plasma samples were collected after an overnight fast and stored at −80°C until analysis. Metabolites were measured using ultra-performance liquid chromatography-tandem mass spectrometry (UPLC-MS/MS, ExionLC™ AD & QTRAP, SCIEX, Framingham, MA), with Multiple Reaction Monitoring (MRM) for quantification. In brief, 40 μL of plasma was mixed with 300 μL of pure methanol, vortexed, and centrifuged (12, 000 rpm, 4°C, 20 min). After refrigeration at −20°C for 30 minutes, the supernatant was centrifuged again (12, 000 rpm, 4°C, 20 min). A 150 μL aliquot of the supernatant was then extracted for analysis. Chromatographic separation was performed on an ACQUITY UPLC HSS T3 C18 column (1.8 µm, 2.1 mm x 100 mm; Waters Corporation, Milford, MA, USA). The analytical conditions were as follows: column temperature, 40°C; flow rate, 0.4 mL/min; injection volume, 2 μL; solvent system, water (0.1% formic acid) and acetonitrile (0.1% formic acid); gradient program, 95:5 V/V at 0 min, 10:90 V/V at 10.0 min, 10:90 V/V at 11.0 min, 95:5 V/V at 11.1 min, 95:5 V/V at 14.0 min. The electrospray ionization source parameters were: source temperature, 500°C; ion spray voltage, 5500 V (positive), −4500 V (negative); ion source gas I, gas II, and curtain gas were set at 55, 60, and 25.0 psi, respectively; the collision gas was set to high. Analyst 1.6.3 software was used for qualitative analysis, and MultiQuant software was employed for integrating and calibrating chromatographic peaks, with the peak area representing the relative content of each metabolite. Quality control samples were injected at regular intervals (every 10 samples) to ensure repeatability. The methods for targeted metabolomic analysis are detailed in Supplementary Material S1.

### Statistical analysis

For the widely targeted metabolomic analysis, both unsupervised PCA (principal component analysis) and supervised orthogonal partial least squares-discrimination analysis (OPLS-DA) with 200-fold permutation test for cross-validation were utilized to identify significant variables. OPLS-DA is a supervised statistical method used to identify differences between groups by maximizing group separation. Variable Importance in Projection (VIP) score helps identify key variables that contribute significantly to the model. Metabolites that showed significant differences between groups were identified based on VIP score of ≥1.0 and an absolute fold change (FC) of ≥1.8 or ≤0.56. Differences in metabolite levels between groups were illustrated using volcano plots and violin plots with the Kruskal-Wallis test. The Kyoto Encyclopedia of Genes and Genomes (KEGG) was employed to annotate, classify, and enrich the differential metabolites and their associated pathways. The ability of each differentiating metabolite to identify frailty was assessed by calculating the area under the receiver operating characteristics (ROC) curve (AUC).

Continuous variables are reported as mean ± standard deviation (SD) or as medians with interquartile ranges (25th-75th percentiles), depending on the distribution. Group comparisons were conducted using analysis of variance (ANOVA) for normally distributed data and the Kruskal-Wallis test for non-normally distributed data. Categorical variables are presented as proportions, with group differences assessed using the Chi-squared test. Spearman’s correlation analysis was performed to explore the associations between continuous levels of GAA and frailty, and related variables, with results visualized in a heatmap. The relationship between continuous GAA levels and the Frailty Index was examined using multivariate linear regression. Multinomial logistic regression was employed to calculate odds ratios (ORs) with 95% confidence intervals (CIs) to assess the odds of prefrailty and frailty associated with GAA levels.

Longitudinal Frailty Index trajectories from 2020 to 2023 were identified using group-based trajectory modeling.^16^ A censored normal model was applied for continuous outcomes. The optimal number (ranging from 1 to 3) and shape of frailty trajectories (linear, quadratic, or cubic) were determined based on the following criteria: (1) the lowest Bayesian Information Criterion (BIC) and Akaike Information Criterion (AIC); (2) each trajectory group comprising at least 5% of participants; and (3) average posterior probabilities for each trajectory group exceeding 0.70.^17^ Ultimately, a model with three distinct frailty trajectories was selected, as detailed in Table S2.

All statistical analyses were conducted using Stata/MP 17.0 (StataCorp LLC, College Station, TX) and R (version 4.1.3) software (R Foundation for Statistical Computing, Vienna, Austria). A two-sided *P*-value of less than 0.05 was considered statistically significant.

## Results

### Metabolome profiling for frailty

**Figure 2.**
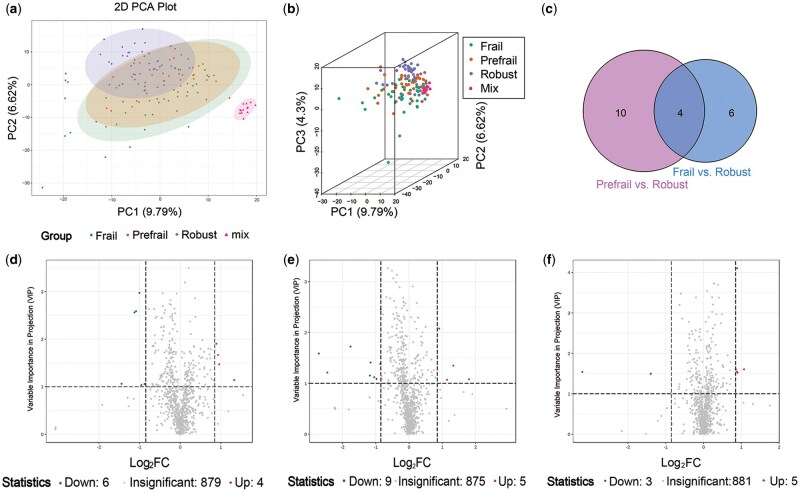
Metabolomic profiling and differential metabolite analysis for frailty: PCA, Venn Diagram, and Volcano Plots. a-b: 2D and 3D PCA plots; c: Venn diagram of common altered metabolites in frail vs. robust and prefrail vs. robust groups; d-f: Volcano plots of differential metabolites in frail vs. robust, prefrail vs. robust, and frail vs. prefrail groups.

The characteristics of the 120 subjects included in the metabolomic profiling, with 40 participants in each of the frail, prefrail, and robust groups, are detailed in Table S3. The groups were comparable in terms of age, gender, comorbidities, BMI, FPG, blood lipids, creatinine, and uric acid levels (all *P* > .05). UPLC-MS/MS analysis identified 889 metabolites across 46 categories. General PCA plots showed a moderate degree of separation between subjects based on their frailty status, as shown in Figure 2a-b.

### Differential metabolites of frailty

To identify differential metabolites associated with frailty, we conducted pairwise comparisons between the robust, prefrail, and frail groups. The corresponding PCA and OPLS-DA plots, along with permutation tests, are presented in Figures S1-3. Using the criteria of FC ≥1.8 or ≤0.56 and VIP ≥1, 10 differential metabolites were identified between the robust and frail groups, with four up-regulated and six down-regulated. Fourteen metabolites were identified between the prefrail and robust groups, and 8 between the prefrail and frail groups. The volcano plots are shown in Figure 2d-f. A Venn diagram revealed four common metabolites between the frail vs. robust and prefrail vs. robust comparisons (Figure 2c). Notably, the levels of guanidinoacetic acid (GAA), 3-indolepropionic acid, and 4-methoxyphenylacetic acid were significantly lower in prefrail and frail subjects, whereas D-urobilinogen levels were significantly higher in those subjects (all *P* < .05). Violin plots illustrating the log-transformed levels of four differentiated metabolites associated with frailty in Figure S4. KEGG pathway enrichment analysis revealed that the differential metabolites between frailty and robustness may be involved in pathways related to arginine and proline metabolism (Figure S5).

Among the differential metabolites, GAA showed the best performance in the ROC analysis, with an AUC of 0.813 for the identification of frailty. Since GAA levels exhibited prominent alterations and hold potential for identifying frail status, we performed targeted metabolomics analysis to precisely quantify GAA levels and validate the findings from the widely targeted analysis.

### Measurement and validation of GAA levels

Targeted metabolomics analysis was employed to validate GAA levels in a larger cross-sectional dataset. GAA levels were significantly lower in the prefrailty (2.1 [1.7-2.5] µmol/L) and frailty (1.8 [1.5-2.2] µmol/L) groups compared to the robust group (2.2 [1.9-2.7] µmol/L) (*P* < .001). The AUC of GAA for identifying frailty in this cohort was 0.670. Characteristics of participants according to quartiles of GAA levels are summarized in Table 1. A total of 1,972 participants were included, with an average age of 72.5 years, and 40.1% were male. Several variables were lower in subjects with higher GAA levels, including age, prevalence of hypertension, diabetes, malnutrition risk, cognitive impairment, comorbidities, polypharmacy, frailty (as defined by Fried’s phenotype and Frailty Index), as well as levels of SBP, HDL, TG, FPG, HbA1c, and TUG duration. Conversely, the percentage of males, levels of DBP, grip strength, gait speed, creatinine, uric acid, and homocysteine, tended to be higher in subjects with higher GAA levels (all *P* < .05).

**Table 1.** Characteristics of subjects by GAA quartiles in 2020.

Variables	Total N = 1972	GAA levels (µmol/l)				*P* value
		Q1(≤1.77) N = 494	Q2 (1.77-2.12) N = 491	Q3 (2.12-2.55) N = 494	Q4 (≥2.55) N = 493	
**General characteristics & lifestyle factors**						
Age (x ± s)	72.5 ± 7.1	74.1 ± 7.9	72.4 ± 7.0	72.0 ± 6.7	71.5 ± 6.6	**<.001**
Male (N, %)	791 (40.1%)	97 (19.6%)	113 (23.0%)	205 (41.5%)	376 (76.3%)	**<.001**
Regular exercise (N, %)	503 (25.5%)	130 (26.4%)	139 (28.4%)	120 (24.3%)	114 (23.1%)	.25
MNA-SF score	13 (12, 14)	12 (11, 14)	13 (11, 14)	13 (12, 14)	13 (12, 14)	**.008**
**Medical condition**						
Hypertension (N, %)	1237 (62.7%)	328 (66.4%)	322 (65.6%)	296 (59.9%)	291 (59.0%)	**.028**
Diabetes (N, %)	633 (32.1%)	219 (44.3%)	148 (30.1%)	122 (24.7%)	144 (29.2%)	**<.001**
Cognitive impairment (N, %)	332 (16.8%)	103 (20.9%)	97 (19.8%)	72 (14.6%)	60 (12.2%)	**<.001**
Comorbidity (N, %)	1133 (57.5%)	323 (65.4%)	289 (58.9%)	261 (52.8%)	260 (52.7%)	**<.001**
Polypharmacy (N, %)	577 (29.3%)	194 (39.3%)	138 (28.1%)	113 (22.9%)	132 (26.8%)	**<.001**
Fried’s criteria						
Robust (N, %)	714 (36.2%)	125 (25.3%)	172 (35.0%)	191 (38.7%)	226 (45.8%)	**<.001**
Prefrailty (N, %)	1088 (55.2%)	293 (59.3%)	276 (56.2%)	274 (55.5%)	245 (49.7%)	
Frailty (N, %)	170 (8.6%)	76 (15.4%)	43 (8.8%)	29 (5.9%)	22 (4.5%)	
Frailty Index	0.14 (0.09, 0.20)	0.16 (0.11, 0.25)	0.15 (0.10, 0.21)	0.13 (0.09, 0.18)	0.11 (0.08, 0.18)	**<.001**
**Physical exam**						
BMI (kg/m^2^)	24.6 (22.6, 26.4)	24.5 (22.6, 26.4)	24.5 (22.7, 26.6)	24.8 (22.5, 26.7)	24.6 (22.6, 26.2)	.83
SBP (mmHg)	137 (126, 148)	138 (129, 149.5)	139 (127, 150)	137 (127, 148)	136 (124, 146)	**<.001**
DBP (mmHg)	80 (72, 87)	78 (70, 85)	81 (73, 87)	81 (73, 87)	81 (75, 89)	**<.001**
Grip strength (kg)	23.4 (18.0, 30.7)	20.0 (16.0, 24.0)	21.5 (17.7, 26.1)	24.1 (19.0, 30.8)	31.7 (24.3, 38.0)	**<.001**
Gait speed (m/s)	0.85 (0.75, 0.96)	0.81 (0.69, 0.93)	0.84 (0.75, 0.95)	0.88 (0.77, 0.98)	0.88 (0.77, 0.98)	**<.001**
TUG (seconds)	10.3 (9.2, 12.0)	10.9 (9.5, 13.2)	10.4 (9.4, 12.0)	10.0 (9.0, 11.6)	10.1 (9.0, 11.5)	**<.001**
**Laboratory data**						
Creatinine (μmol/L)	66 (55, 80)	63 (53, 78)	63 (53, 77)	66 (55, 79)	72 (62, 84)	**<.001**
Uric acid (μmol/L)	366 (311, 426)	359 (305, 407)	359 (306, 423)	365 (309, 431)	381 (325, 436)	**<.001**
HDL-C (mmol/L)	1.2 (1.0, 1.4)	1.2 (1.0, 1.4)	1.2 (1.0, 1.5)	1.2 (1.0, 1.4)	1.1 (1.0, 1.3)	**<.001**
LDL-C (mmol/L)	2.7 (2.1, 3.4)	2.7 (2.1, 3.4)	2.8 (2.1, 3.4)	2.7 (2.1, 3.5)	2.8 (2.1, 3.4)	.79
TC (mmol/L)	5.3 (4.5, 6.0)	5.2 (4.3, 6.1)	5.3 (4.6, 6.1)	5.3 (4.5, 6.1)	5.2 (4.5, 5.9)	.13
TG (mmol/L)	1.5 (1.0, 2.0)	1.5 (1.1, 2.0)	1.5 (1.1, 2.0)	1.5 (1.1, 2.1)	1.4 (1.0, 1.9)	**.048**
FPG (mmol/L)	5.9 (5.5, 6.8)	6.1 (5.5, 7.7)	5.9 (5.4, 6.6)	5.9 (5.5, 6.6)	5.9 (5.5, 6.7)	**<.001**
HbA1c (%)	5.8 (5.4, 6.4)	6.0 (5.5, 6.8)	5.8 (5.3, 6.2)	5.7 (5.3, 6.2)	5.8 (5.4, 6.4)	**<.001**
Homocysteine (μmol/L)	8.4 (5.8, 11.2)	8.6 (5.6, 11.6)	8.1 (5.6, 10.7)	8.2 (5.7, 11.0)	8.8 (6.3, 11.9)	**.026**

GAA: guanidinoacetic acid, Q: quartiles, MNA-SF: mini-nutritional assessment-short form, BMI: body mass index, SBP: systolic blood pressure, DBP: diastolic blood pressure, TUG: timed up and go test, HDL-C: high-density lipoprotein cholesterol, LDL-C: low-density lipoprotein cholesterol, TG: triglycerides, TC: total cholesterol, FPG: fasting plasma glucose, HbA1c: hemoglobin A1c. Regular exercise as expenditure of physical activity per week <383 kcal for men, <270 kcal for women. Comorbidity was defined as the coexistence of ≥ 2 chronic conditions, and polypharmacy as the use of ≥ 5 categories of medication. Age was presented as mean ± standard deviation (SD) and compared among groups using ANOVA. Other continuous variables were presented as median (Interquartile range, IQR) and compared using the Kruskal-Wallis test. Categorical variables were presented as proportions, with group differences assessed by the Chi-squared test. Bold values indicate statistically significant differences (p < 0.05).

### Association between GAA levels and frailty status

We further investigated the association of frailty and its related factors with GAA levels. GAA levels were negatively associated with Fried’s phenotype (r_s_ = −0.197), the Frailty Index (r_s_ = −0.256), TUG, and age, and positively associated with grip strength, gait speed, creatinine, uric acid levels, as well as MNA-SF score (all *P* < .01). These relationships are illustrated in a heatmap (Figure S6). Multinomial logistic regression revealed that in the fully adjusted model, higher GAA levels were significantly associated with lower odds of prefrailty and frailty according to Fried’s criteria. Specifically, for GAA quartiles Q2-Q4, the odds ratios (ORs) for prefrailty were 0.61 (95% CI: 0.44-0.85), 0.64 (95% CI: 0.46-0.89), and 0.46 (95% CI: 0.32-0.66), respectively, and for frailty, the ORs were 0.40 (95% CI: 0.21-0.75), 0.29 (95% CI: 0.14-0.59), and 0.15 (95% CI: 0.07-0.33), *P* < .01 for all associations (Model 2 in Table 2). Continuous GAA levels were inversely associated with Frailty Index in multivariate linear regression analysis after adjusting for covariates (Coefficient β = −0.022, 95% CI: −0.027 to −0.017, *P* < .001), as shown in Table S4.

**Table 2.** Multinomial logistic regression for the association of GAA levels with frailty in 2020 (N = 1972).

Variables	Model 1 ORs (95% CI)	*P* value	Model 2 ORs (95% CI)	*P* value
**Prefrailty**				
Continuous GAA (μmol/L)	0.60 (0.49, 0.73)	**<.001**	0.62 (0.50, 0.76)	**<.001**
GAA quartiles				
Q1 (≤1.77 µmol/l)	Reference		Reference	
Q2 (1.77-2.12 µmol/l)	0.58 (0.42, 0.80)	**.001**	0.61 (0.44, 0.85)	**.004**
Q3 (2.12-2.55 µmol/l)	0.61 (0.44, 0.84)	**.003**	0.64 (0.46, 0.89)	**.009**
Q4 (≥2.55 µmol/l)	0.44 (0.31, 0.62)	**<.001**	0.46 (0.32, 0.66)	**<.001**
**Frailty**				
Continuous GAA (μmol/L)	0.23 (0.15, 0.36)	**<.001**	0.26 (0.16, 0.41)	**<.001**
GAA quartiles				
Q1 (≤1.77 µmol/l)	Reference		Reference	
Q2 (1.77-2.12 µmol/l)	0.30 (0.17, 0.55)	**<.001**	0.40 (0.21, 0.75)	**.004**
Q3 (2.12-2.55 µmol/l)	0.25 (0.13, 0.47)	**<.001**	0.29 (0.14, 0.59)	**.001**
Q4 (≥2.55 µmol/l)	0.13 (0.06, 0.26)	**<.001**	0.15 (0.07, 0.33)	**<.001**

Model 1: adjusted for age, gender, exercise, BMI, and nutritional status; Model 2: adjusted for model 1 covariates plus mean blood pressure, FPG, dyslipidemia, and cognitive ability. GAA: guanidinoacetic acid, Q: quartiles, ORs: odds ratios, CI: confidence interval. Bold values indicate statistically significant differences (p < 0.05).

### Association between GAA levels and frailty progression

Over a 3-year follow-up period from 2020 to 2023, group-based trajectory modeling identified three distinct Frailty Index trajectories: low-elevated (59.6%, n = 910), moderate-elevated (34.1%, n = 521), and high-elevated (6.3%, n = 97) (Figure S7). The 3 frailty trajectories represented groups with aggravated frailty status over time, categorized by their baseline Frailty Index as low, moderate, and high, respectively. Compared to those in the low-elevated group, subjects in the moderate- and high-elevated groups were older, less likely to be male, participated less frequently in regular exercise, and had higher prevalence of hypertension, diabetes, and cognitive impairment. They also had lower MNA-SF scores, grip strength, and gait speed, but higher BMI, homocysteine levels, and took longer to complete the TUG test (*P* < .001 or *P* = .015; Table S5). Multinomial logistic regression analysis indicated that, in the fully adjusted model, compared to the first quartile of GAA levels, subjects in the highest quartile had a significantly lower odds of following the moderate- and high-elevated frailty trajectories (OR = 0.64, 95% CI: 0.44-0.92, and OR = 0.34, 95% CI: 0.13-0.85, respectively; *P* = .016 and *P* = .022), as shown in Table 3.

**Table 3.** Multinomial logistic regression for the association between GAA levels and Frailty Index trajectories (N = 1528).

Variables	Frailty Index trajectories					
	Low-elevated ORs (95% CI)	*P* value	Moderate-elevated ORs (95% CI)	*P* value	High-elevated ORs (95% CI)	*P* value
**Continuous GAA levels**						
GAA (μmol/L)	Base outcome	-	0.76 (0.60, 0.96)	**0.022**	0.40 (0.23, 0.72)	**.002**
**GAA quartiles (μmol/L)**						
Q1 (≤ 1.792)	Base outcome	-	Reference	-	Reference	-
Q2 (1.792∼2.133)	Base outcome	-	0.84 (0.59, 1.19)	0.323	0.67 (0.33, 1.36)	0.268
Q3 (2.133∼2.572)	Base outcome	-	0.78 (0.52, 1.15)	0.210	0.44 (0.19, 0.60)	**.011**
Q4 (≥ 2.572)	Base outcome	-	0.64 (0.44, 0.92)	**0.016**	0.34 (0.13, 0.85)	**.022**

The models were adjusted for age, gender, exercise, BMI, nutritional status, mean blood pressure, FPG, dyslipidemia, and cognitive ability. GAA: guanidinoacetic acid, Q: quartiles, ORs: odds ratios, CI: confidence interval. Bold values indicate statistically significant differences (p < 0.05).

### Subgroup and sensitivity analysis

Longitudinal subgroup analysis showed that the association between continuous GAA levels and moderate-elevated, high-elevated trajectories of frailty are particularly pronounced in participants over 71 years old, female, and those with creatinine levels <66 μmol/L, with notable gender interactions (Table S6). There were no significant interactions in cross-sectional subgroup analysis (Figure S8). Sensitivity analysis identified binary FI trajectories, classified as high and low, and the results were consistent with the analysis of three trajectories. (Table S7).

## Discussion

This study is the first to identify GAA as a potential metabolic biomarker for frailty by metabolomics approach. We validated our findings in a larger cross-sectional study, confirming that GAA levels were significantly lower in the subjects with frailty, and higher GAA levels are linked to a lower odds of frailty. Additionally, data from an ongoing longitudinal cohort suggest that higher GAA levels predict a lower likelihood of frailty progression.

GAA plays a crucial role as a precursor to creatine, a compound vital for energy storage and muscle function. The synthesis of GAA begins when the enzyme arginine amidinotransferase (AGAT) transfers an amidino group from arginine to glycine, followed by methylation by guanidinoacetate N-methyltransferase using S-adenosylmethionine to produce creatine.^18–20^ Our KEGG enrichment analysis also highlighted alterations in arginine and proline metabolism. The relationship between GAA and frailty is mainly rooted in GAA’s role in creatine synthesis and energy metabolism. Creatine is crucial for energy storage and metabolism in muscle tissue, as it regenerates ATP needed for muscle contraction and overall energy, which can be compromised in frail individuals.^21–22^

Studies directly linking GAA to frailty are limited. An earlier report suggested that serum GAA levels were lower in older subjects compared to middle-aged individuals, and even lower in bedridden older adults with reduced muscle mass.^23^ This aligns with our findings that lower GAA levels are observed in frail older adults. Diet and hormone levels influence AGAT activity, contributing to age-related inhibition and reduced GAA production in older adults.^24^ Imbalances in GAA could negatively impact muscle function and overall energy metabolism, potentially exacerbating frailty in older adults. Evidence suggests that circulating GAA levels increase after exhaustive exercise compared to baseline.^25^

In the interventions for frailty, creatine supplementation, especially when combined with resistance exercise, has been shown to significantly benefit muscle aging.^21^^,^^22^ The favorable outcomes are primarily achieved by enhancing the rapid replenishment of ATP, increasing protein synthesis, optimizing energy availability, and exerting anti-inflammatory effects. Although GAA supplementation is more commonly used in animal studies and has been associated with potential side effects including hyperhomocysteinemia and brain choline depletion,^26^ additional GAA supplementation has shown positive effects independent of creatine alone. The underlying mechanism may involve GAA supplementation reducing systemic inflammatory responses and lipid deposition, both of which are closely linked to frailty.^27–29^ In older populations, particularly those with vegetarian diets that reduce the body’s creatine pool, GAA supplementation could help address this deficiency.^26^^,^^30^ GAA supports skeletal muscle growth, spares arginine for protein synthesis, and stimulates insulin, an anti-catabolic hormone that helps prevent muscle breakdown. The storage of creatine in some energy-demanding tissues is not infinite,^31^ GAA may serve as a superior alternative for optimizing energy metabolism in tissues requiring high energy, such as skeletal muscle, brain, and myocardium.

A creatine-GAA mixture appears to be more effective than creatine alone in boosting tissue creatine content, improving upper body strength and brain bioenergetics in older adults,^32^ and augmenting extracellular mass, and enhances cerebral oxygen saturation in a cognitive task in younger populations.^33^^,^^34^ This mixture is generally well-tolerated in healthy adults and mitigates the risk of increased homocysteine levels related to GAA alone.^35^^,^^36^ The association between higher GAA levels and lower probabilities of frailty suggests that a creatine-GAA combination could be considered as a novel energy-boosting strategy or as a follow-up supplementation for those who have completed traditional muscle creatine-loading protocols. However, further research is needed to confirm the efficacy and safety of GAA supplementation in older adults.

Considering the potential gender-specific effects on GAA levels, we conducted a subgroup analysis based on gender. The cross-sectional association between GAA and frailty was consistent across both genders, indicating the stability of this relationship. Longitudinally, the association was significant in females, older individuals, and those with lower creatinine levels, with a noticeable interaction effect between genders. These populations share the characteristic of having lower GAA levels compared to their counterparts, indicating a more pronounced relationship between reduced GAA levels and higher frailty odds.

A key strength of our study is the combination of widely targeted and targeted metabolomics. Widely targeted metabolomics allowed broad screening of metabolites associated with frailty, while targeted metabolomics enabled precise quantification of candidates. This two-step approach improved the efficiency and robustness of biomarker discovery. Furthermore, we validated our findings in large cohorts, both cross-sectionally and longitudinally, enhancing the reliability and generalizability of the results. However, this study has limitations. First of all, we cannot completely rule out the impact of unmeasured confounding factors, although we carefully considered covariates that might influence GAA levels, such as malnutrition and exercise. Additionally, our study subjects may represent a relatively healthy older population, as they were proactive in visiting community health service centers and willing to participate in follow-up investigations. Therefore, caution is needed when generalizing these results to other populations. Lastly, the trajectory groups are mainly defined by baseline Frailty Index scores, which could influence its association with GAA. Also, the short follow-up period may not fully capture the long-term effects of GAA on frailty progression. Future studies with longer follow-up and larger sample sizes could offer more insights into the long-term impact of GAA on frailty trajectories.

The findings of this study have practical clinical significance by providing evidence that plasma GAA levels could facilitate the early identification of relatively healthy, community-dwelling older individuals at higher likelihood of developing frailty. Our results provide evidence for implementing precise nutritional interventions, such as GAA or creatine-GAA mixture supplementation, prior to the onset of frailty. Future studies are needed to confirm these results and clarify the pathological mechanisms underlying the association between GAA and frailty.

In conclusion, this study demonstrates that plasma GAA is a novel metabolic biomarker associated with frailty in older adults. Higher GAA levels are not only linked to lower odds of frailty but also predict a lower likelihood of frailty progression over time. GAA can serve as valuable indicators of frailty and aid in developing targeted treatment strategies.

## Supplementary Material

glaf127_Supplementary_Data
