# Supplementary material for: Metabolomics-based identification and validation of the creatine precursor guanidinoacetic acid for frailty in older adults
Source: J Gerontol A Biol Sci Med Sci. 2025 Jun 11;80(10):glaf127. doi: 10.1093/gerona/glaf127 (PMC12462772; doi:10.1093/gerona/glaf127)
Supplement: glaf127_Supplementary_Data [file glaf127_supplementary_data.pdf]

Metabolomics-based identification and validation of creatine precursor guanidinoacetic acid for frailty in older adults

Yin Yuan, PhD#, Xiaoming Huang, MS#, Siyang Lin, PhD, Wenwen Lin, MS, Feng Huang, MS*, and Pengli Zhu, MS*

Contents:

eMethods 1 The method for targeted metabolomic analysis
eTable 1 List of 40 variables included in the Frailty Index
eTable 2 The Maximum Likelihood Estimates from the Censored Normal Model
eTable 3 Characteristics of participants in metabolome profiling for frailty (N=120)
eTable 4 Association of GAA levels and frailty index in 2020 (N=1972)
eTable 5 Characteristics of subjects by the trajectories of Frailty Index (2020 - 2023)
eTable 6 Subgroup analysis for frailty trajectories according to the continuous GAA levels from 2020 to 2023 (N=1528)
eTable 7 Association between low- and high-elevated frailty trajectories and GAA levels (N=1528)

eFigure 1 Plasma metabolomics profiling for frail vs. robust group
eFigure 2 Plasma metabolomics profiling for prefrail vs. robust group
eFigure 3 Plasma metabolomics profiling for frail vs. prefrail group
eFigure 4 Violin plots for the log-transformed four differentiated metabolites of frailty
eFigure 5 KEGG pathway enrichment for differential metabolites of frailty
eFigure 6 Heatmap of frailty and its related factors with GAA levels
eFigure 7 The trajectory of the Frailty Index over a three-year follow-up (2020–2023)
eFigure 8 Subgroup analysis of the association between the highest quartile of guanidinoacetic acid (GAA) and prefrailty and frailty (N=1972)

eMethods 1 The method for targeted metabolomic analysis

Plasma GAA levels were determined using UPLC-MS/MS. Specifically, 100 μL of plasma was transferred to a 1.5 mL Eppendorf tube, and 5 μL of internal standard (d2-GAA at 100 μmol/L) was added. To precipitate proteins, 450 μL of acetonitrile was added and the mixture was vortexed. After centrifugation (13,000 rpm, 4°C, 15 min), 10 μL of the supernatant was injected into the UPLC-MS system (ACQUITY UPLC I-Class/Xevo TQD, Waters, Milford, USA). Chromatographic separation was performed on a CORTECS HILIC column (1.6 μm, 2.1 mm × 100 mm, Waters, USA) at 40°C with a flow rate of 400 μL/min. The mobile phase A consisted of acetonitrile/H2O (95:5) with 1 mmol/L ammonium acetate and 0.1% formic acid, while phase B was acetonitrile/H2O (50:50) with the same additives. The gradient started at 90% A for 0.5 min, decreased to 30% A over 3 min, and held at 30% A for 0.5 min before returning to 90% A. The column was equilibrated for 2.5 min. In positive ionization mode, the capillary voltage was set to 0.5 kV, with a desolvation temperature of 550°C and a source temperature of 120°C. Stable isotope-labeled guanidineacetic acid (d2-GAA) served as the internal standard. Multiple reaction monitoring (MRM) transitions used were d2-GAA 120>103 and GAA 118>101. GAA quantification was based on a calibration curve of the peak area ratio of analyte to internal standard.

eTable 1 List of 40 variables included in the Frailty Index

Category	Variables	Definition
Basic/instrumental activities of daily living (ADL/IADL)	Bathing	Independence = 0, Need help = 1
	Dressing	Independence = 0, Need help = 1
	Toileting	Independence = 0, Need help = 1
	Transferring	Independence = 0, Need help = 1
	Continence	Independence = 0, Need help = 1
	Feeding	Independence = 0, Need help = 1
	Use Telephone	Independence = 0, Need help = 1
	Shopping	Independence = 0, Need help = 1
	Food Preparation	Independence = 0, Need help = 1
	Housekeeping	Independence = 0, Need help = 1
	Laundry	Independence = 0, Need help = 1
	Transportation	Independence = 0, Need help = 1
	Responsibility for own medications	Independence = 0, Need help = 1
	Handle Finances	Independence = 0, Need help = 1
Physical health and medical conditions	Self rating of health	Poor = 1, Fair = 0.75, Good = 0.5, very good = 0.25, Excellent = 0
	Hypertension	No = 0, Yes = 1
	Diabetes	No = 0, Prediabetes = 0.5, Yes = 1
	Coronary heart disease	No = 0, Yes = 1
	Heart failure	No = 0, Yes = 1
	Cancer	No = 0, Yes = 1
	Stroke	No = 0, Yes = 1
	Chronic respiratory disease	No = 0, Yes = 1
	Chronic kidney diseases	No = 0, Yes = 1
Geriatric syndrome	Polypharmacy	No = 0, Yes = 1
	Malnutrition	MNA-SF score ≥12 = 0, <12 = 1
	Exercise (expenditure of physical activity per week < 383 kcal for men, < 270 kcal for women)	Meet the standard[1] = 0, not = 1
	Vision	Normal = 0, mild impaired = 0.5, impaired = 1
	Hearing	Normal = 0, mild impaired = 0.5, impaired = 1
	Persistent fatigue	No = 0, Yes = 1
	Balance	TUG <10s =0,10~20s=0.5, >20s=1
	Incontinence	No = 0, Occasionally = 0.5, Yes = 1
	History of fall	No = 0, one time = 0.5, ≥ 2 times or severely injured = 1
	Anxiety	“GAD-7≤ 4” = 0, “GAD-7 5~13” = 0.5, “GAD-7≥14” = 1
	Depression	“GDS-4=0”=0, “GDS-4=1” = 0.5, “GDS-4≥2” =1
	Sleep	“AIS<4” = 0, “AIS 4~5” = 0.5, “AIS ≥ 6” = 1
	Chronic pain	No = 0, Yes = 1

eTable 1 List of 40 variables included in the Frailty Index (continued)

Category	Variables	Definition
	Cognition	“Minicog >3” =0, “Minicog =3” =0.5, “Minicog ≤2” =1
	Grip strength	Fried’s standard
	Gait speed	Fried’s standard
	Family support	SSRS ≥11=0, <11 =1

Note: MNA-SF: mini nutritional assessment-short form, GAD-7: generalized anxiety disorder scale-7, GDS-4: geriatric depression scale-4, AIS: Athens insomnia scale, TUG: timed up and go test, SSRS: social support rating scale.

eTable 2 The Maximum Likelihood Estimates from the Censored Normal Model

Group	Parameter	Estimate	Standard Error	T for H0: Parameter=0	Prob > |T|
1	Intercept	-0.46093	0.01690	-27.269	<0.001
	Linear	0.79632	0.01651	48.233	<0.001
	Quadratic	-0.14903	0.00435	-34.237	<0.001
2	Intercept	-0.51877	0.01089	-47.651	<0.001
	Linear	0.76037	0.01263	60.191	<0.001
	Quadratic	-0.13797	0.00318	-43.357	<0.001
3	Intercept	-0.10775	0.05424	-1.987	0.0470
	Linear	0.57786	0.05410	10.681	<0.001
	Quadratic	-0.10920	0.01303	-8.378	<0.001
	Sigma	0.06350	0.00089	71.026	<0.001
**Group membership**					
	Group 1 (%)	34.09686	5.64405	6.511	<0.001
	Group 2 (%)	59.55497	6.54764	8.640	<0.001
	Group 3 (%)	6.34817	1.32929	5.026	<0.001

eTable 3 Characteristics of participants in metabolome profiling for frailty (N=120)

Variables	Frailty (N=40)	Prefraity (N=40)	Robustness (N=40)	*P* value
Age ($\bar{x}$±s)	78.7 ± 7.4	77.4 ± 7.2	77.2 ± 4.9	0.56
Female (N, %)	27 (68%)	28 (70%)	23 (58%)	0.56
Comorbidity (N, %)	17 (43%)	15 (38%)	14 (35%)	0.84
BMI (kg/m^2)	23.8 (22.0, 25.6)	24.4 (22.2, 26.4)	25.1 (23.0, 26.7)	0.21
FPG (mmol/L)	5.5 (5.1, 6.1)	5.7 (5.2, 6.2)	5.8 (5.3, 6.6)	0.35
TC (mmol/L)	5.4 ±1.3	5.4 ±1.1	5.5 ±1.0	0.90
TG (mmol/L)	1.4 (1.1, 1.9)	1.3 (1.0, 1.8)	1.4 (1.1, 2.0)	0.73
HDL-C (mmol/L)	1.2 (1.0, 1.5)	1.2 (1.0, 1.6)	1.1 (1.0, 1.3)	0.21
LDL-C (mmol/L)	3.0 ±1.1	2.9 ±1.0	3.0 ± 0.9	0.82
Uric acid (μmol/L)	381 (302, 430)	376.5 (300, 413)	363 (306, 421)	0.96
Creatinine (μmol/L)	67.5 (59.5, 89.0)	66.5 (53.5, 88.9)	67.0 (54.0, 77.0)	0.57
Fried's phenotype	3 (3, 4)	2 (1, 2)	0 (0, 0)	**<0.001**
Frailty index	0.29 (0.26, 0.34)	0.20 (0.13, 0.22)	0.06 (0.05, 0.08)	**<0.001**

Note: BMI: body mass index, FPG: fasting plasma glucose, TC: total cholesterol, TG: triglycerides, HDL-C: high-density lipoprotein cholesterol, LDL-C: low-density lipoprotein cholesterol. Comorbidity was defined as the coexistence of ≥2 chronic conditions.

eTable 4 Association of GAA levels and frailty index in 2020 (N=1972)

**Variables**	**Coefficient β (95% CI)**	***P* value**
GAA	-0.022 (-0.027, -0.017)	**<0.001**
Age	0.003 (0.003, 0.004)	**<0.001**
BMI	0.004 (0.003, 0.005)	**<0.001**
MNA-SF score	-0.019 (-0.021, -0.017)	**<0.001**
SBP	0.0002 (0.00002, 0.0004)	**0.035**
Creatinine	0.0002 (0.0007, 0.0003)	**0.002**
TG	0.004 (0.001, 0.007)	**0.009**
TC	-0.005 (-0.009, -0.003)	**<0.001**
FPG	0.005 (0.004, 0.007)	**<0.001**
Minicog score	-0.005 (-0.008, -0.002)	**0.001**
_cons	0.047(-0.01, 0.104)	0.106

Note: The model was adjusted for age, creatinine, BMI, MNA-SF score, SBP, TG, TC, LDL-C, HDL-C, FPG, and minicog score. GAA: guanidinoacetic acid, BMI: body mass index, MNA-SF: mini-nutritional assessment-short form, SBP: systolic blood pressure, TG: triglycerides, TC: total cholesterol, FPG: fasting plasma glucose.

eTable 5 Characteristics of subjects by the trajectories of Frailty Index (2020 - 2023)

Variables	Total N=1528	Frailty Index trajectories			*P* value
		**Low-elevated (N=910)**	**Moderate-elevated (N=521)**	**High-elevated (N=97)**	
Age (year, $\bar{x}$±s)	72.1 ± 6.8	70.4 ± 5.8	73.8 ± 7.0	79.8 ± 7.7	**<0.001**
Male (N, %)	615 (40.2%)	412 (45.3%)	181 (34.7%)	22 (22.7%)	**<0.001**
Regular exercise (N, %)	1140 (74.6%)	754 (82.9%)	348 (66.9%)	38 (39.6%)	**<0.001**
MNA-SF score	13 (12, 14)	13 (12, 14)	12 (11, 14)	11 (10, 12)	**<0.001**
BMI (kg/m^2)	24.6 (22.7, 26.5)	24.4 (22.6, 26.2)	24.9 (23.0, 26.9)	25.1 (21.8, 27.0)	**0.015**
Grip strength (kg)	24 (18.8, 31.2)	25.6 (20.7, 34.0)	22.0 (17.3, 28.0)	17.0 (13.0, 22.0)	**<0.001**
Gait speed (m/s)	0.86 (0.76, 0.97)	0.91 (0.81, 0.99)	0.81 (0.72, 0.94)	0.65 (0.50, 0.77)	**<0.001**
TUG (seconds)	10.2 (9.1, 11.7)	9.7 (8.8, 10.9)	10.9 (9.8, 12.8)	14.1 (11.7, 21.3)	**<0.001**
Creatinine (μmol/L)	66.0 (54.0, 79.0)	65.5 (54.0, 78.0)	66.0 (54.0, 80.0)	69.0 (57.0, 85.0)	0.074
Homocysteine (μmol/L)	8.4 (5.8, 11.1)	8.0 (5.7, 10.4)	8.7 (5.9, 11.8)	10.9 (8.5, 13.9)	**<0.001**
Hypertension (N, %)	956 (62.6%)	467 (51.3%)	403 (77.4%)	86 (88.7%)	**<0.001**
Diabetes (N, %)	499 (32.7%)	200 (22.0%)	245 (47.0%)	54 (55.7%)	**<0.001**
Cognitive impairment (N,%)	228 (14.9%)	79 (8.7%)	116 (22.3%)	33 (34.0%)	**<0.001**

Note: MNA-SF: mini-nutritional assessment-short form, BMI: body mass index, TUG: timed up and go test.

eTable 6 Subgroup analysis for frailty trajectories according to the continuous GAA levels from 2020 to 2023 (N=1528)

Variables	No. of participants	Moderate-elevated ORs (95% CI)	*P* value	*P* for interaction	High-elevated ORs (95% CI)	*P* value	*P* for interaction
**Age (yrs)**							
<71	721	0.79 (0.56, 1.13)	0.194	0.807	0.25 (0.06, 1.05)	0.058	0.891
≥71	807	**0.73 (0.54, 1.00)**	**0.049**		**0.41 (0.21, 0.79)**	**0.008**	
**Gender**							
Male	615	0.91 (0.67, 1.24)	0.547	**0.047**	1.03 (0.32, 3.33)	0.957	**0.011**
Female	913	**0.59 (0.41, 0.85)**	**0.004**		**0.22 (0.11, 0.47)**	**<0.001**	
**Creatinine**							
<66μmol/L	747	**0.58 (0.40, 0.82)**	**0.002**	**0.002**	**0.32 (0.14, 0.75)**	**0.009**	0.296
≥ 66μmol/L	781	0.95 (0.69, 1.30)	0.726		0.53 (0.23, 1.24)	0.142	
**BMI**							
<24kg/m^2	631	0.76 (0.52, 1.10)	0.149	0.464	**0.41 (0.18, 0.97)**	**0.041**	0.133
≥ 24kg/m^2	897	0.76 (0.57, 1.02)	0.072		**0.33 (0.14, 0.76)**	**0.009**	
**History of hypertension**							
Yes	956	0.87 (0.65, 1.16)	0.345	0.088	**0.45 (0.23, 0.86)**	**0.015**	0.342
No	572	**0.58 (0.36, 0.94)**	**0.027**		0.30 (0.04, 2.27)	0.246	
**History of diabetes**							
Yes	1029	0.82 (0.57, 1.18)	0.287	0.735	0.53 (0.25, 1.11)	0.092	0.727
No	499	0.83 (0.60, 1.15)	0.266		0.37 (0.13, 1.03)	0.056	

Note: BMI: body mass index, MNA-SF: mini-nutritional assessment-short form.

eTable 7 Association between low- and high-elevated frailty trajectories and GAA levels (N=1528)

**Variables**	**Frailty trajectories**			
	**Low-elevated ORs (95% CI)**	***P* value**	**High-elevated ORs (95% CI)**	***P* value**
**Continuous GAA levels**				
GAA (μmol/L)	Base outcome	-	0.67 (0.49, 0.92)	**0.014**
**GAA quartiles (μmol/L)**				
Q1 (≤1.792)	Base outcome	-	Reference	-
Q2 (1.792~2.133)	Base outcome	-	0.79 (0.51, 1.23)	0.294
Q3 (2.133~2.572)	Base outcome	-	0.60 (0.37, 0.96)	**0.032**
Q4 (≥ 2.572)	Base outcome		0.54 (0.32, 0.92)	**0.023**

Note: The models were adjusted for age, gender, exercise, BMI, nutritional status, mean blood pressure, fasting plasma glucose, dyslipidemia, and cognitive ability.

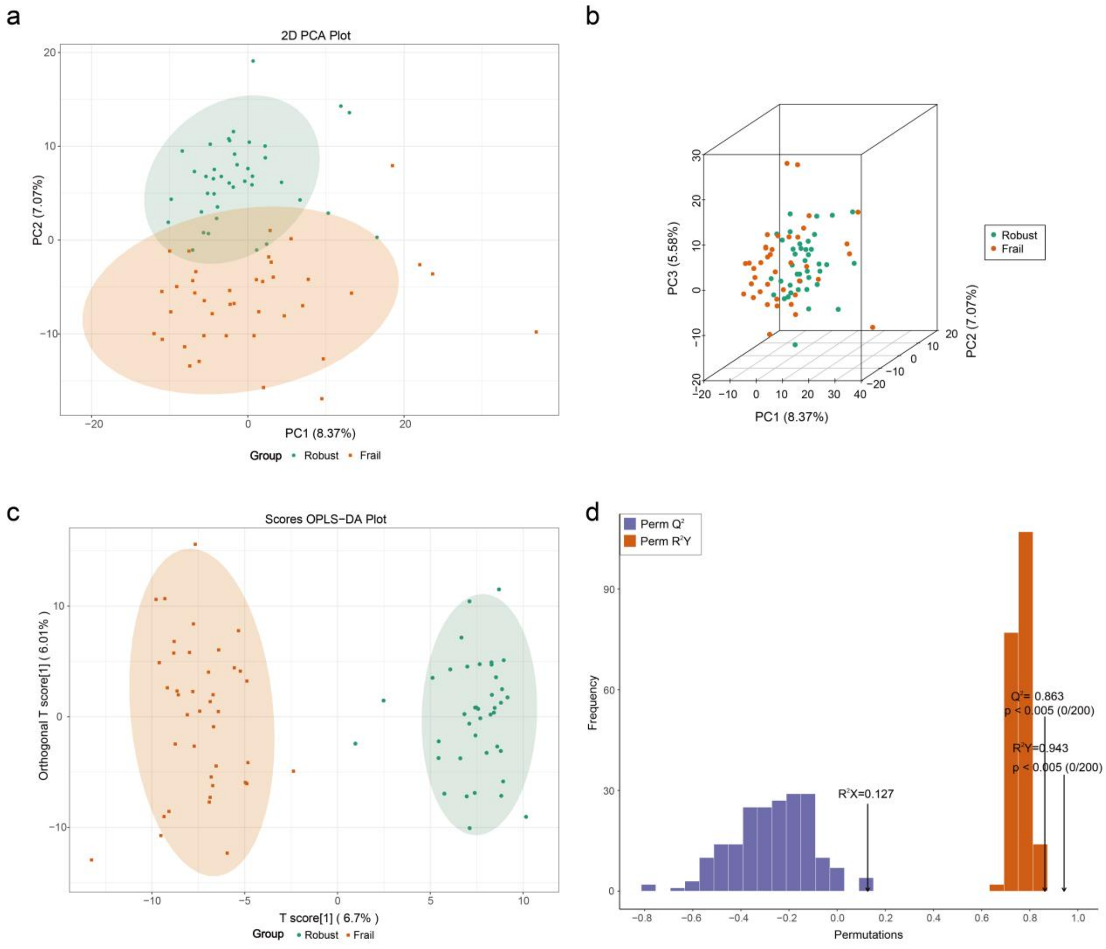

eFigure 1 Plasma metabolomics profiling for frail vs. robust group

Note: a: 2D PCA plot, b: 3D PCA plot, c: OPLS-DA plots, d: 200-fold permutation test.

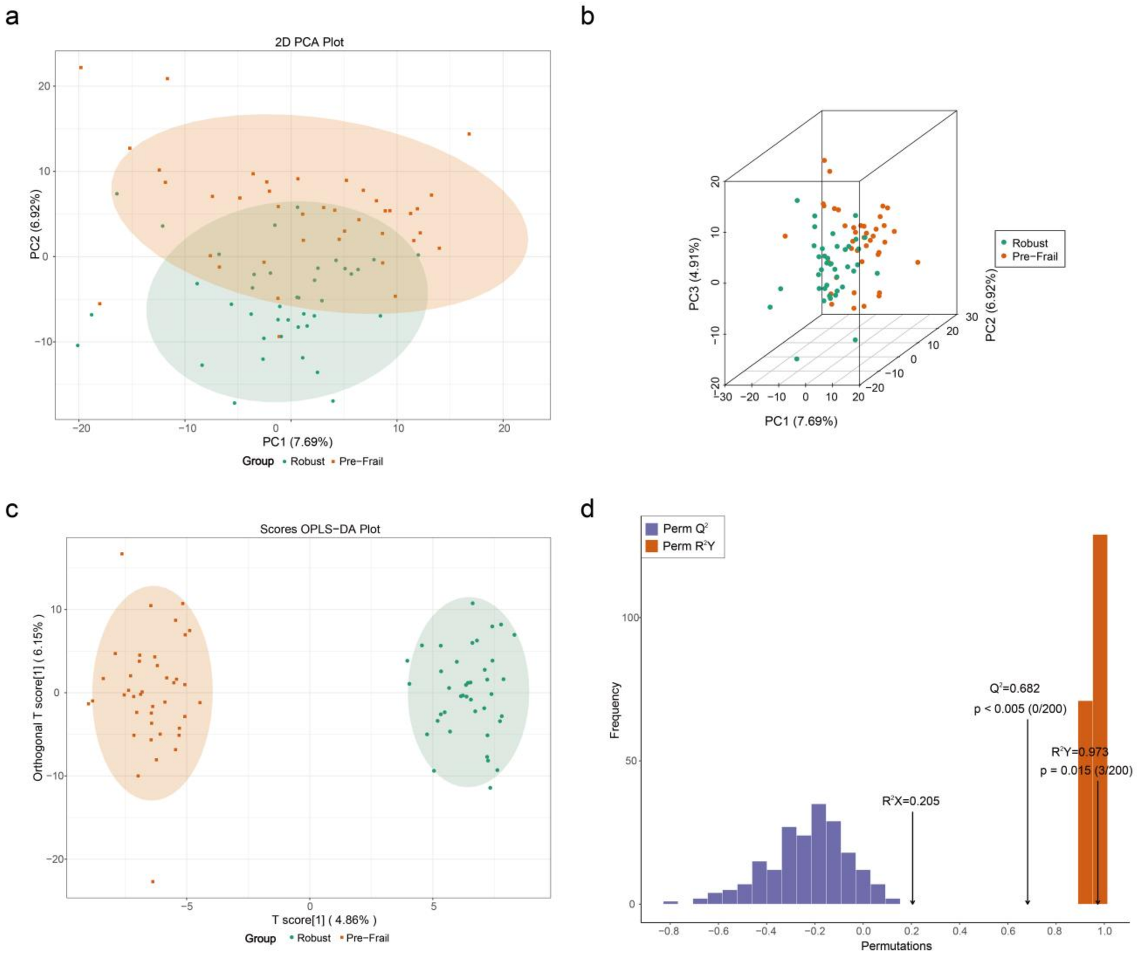

eFigure 2 Plasma metabolomics profiling for prefrail vs. robust group

Note: a: 2D PCA plot, b: 3D PCA plot, c: OPLS-DA plots, d: 200-fold permutation test.

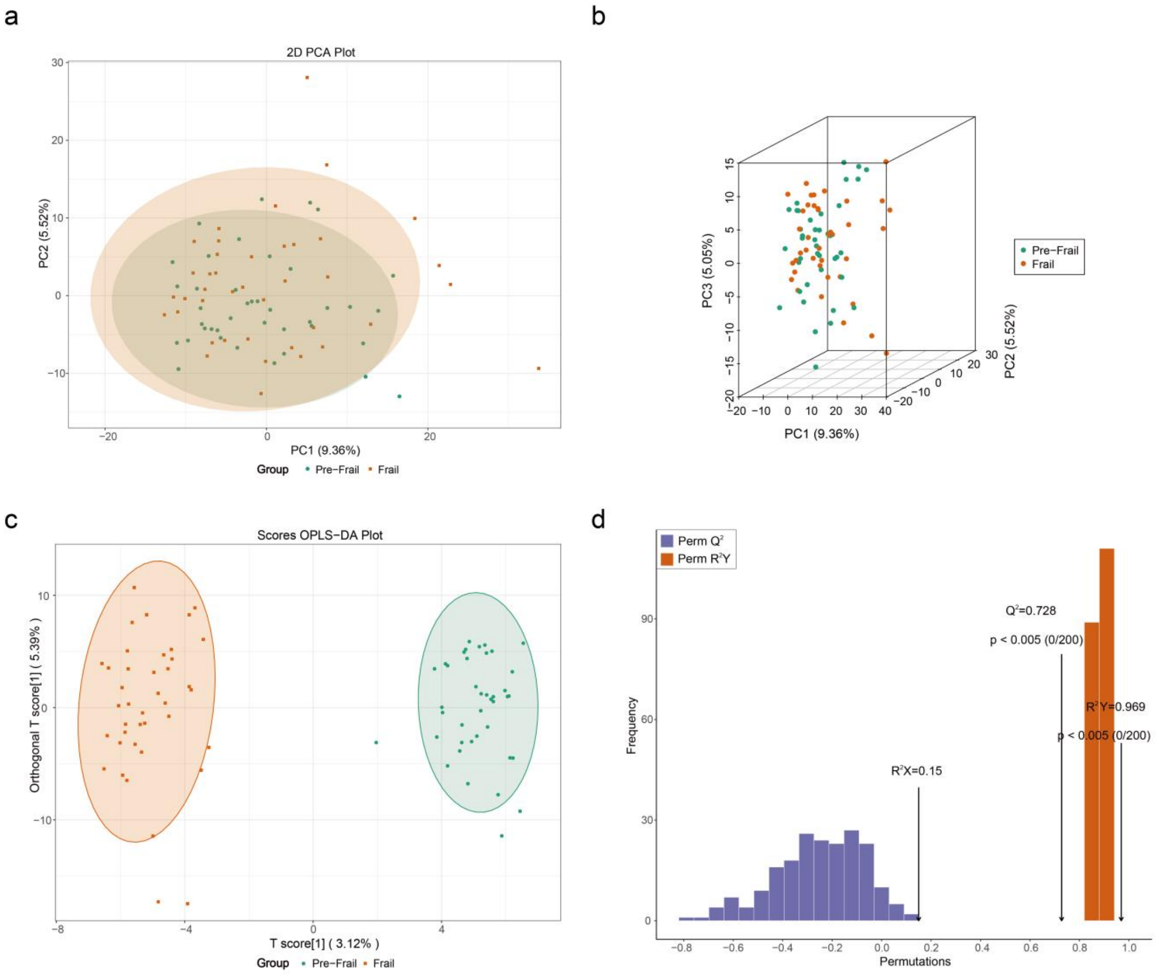

eFigure 3 Plasma metabolomics profiling for frail vs. prefrail group

Note: a: 2D PCA plot, b: 3D PCA plot, c: OPLS-DA plots, d: 200-fold permutation test.

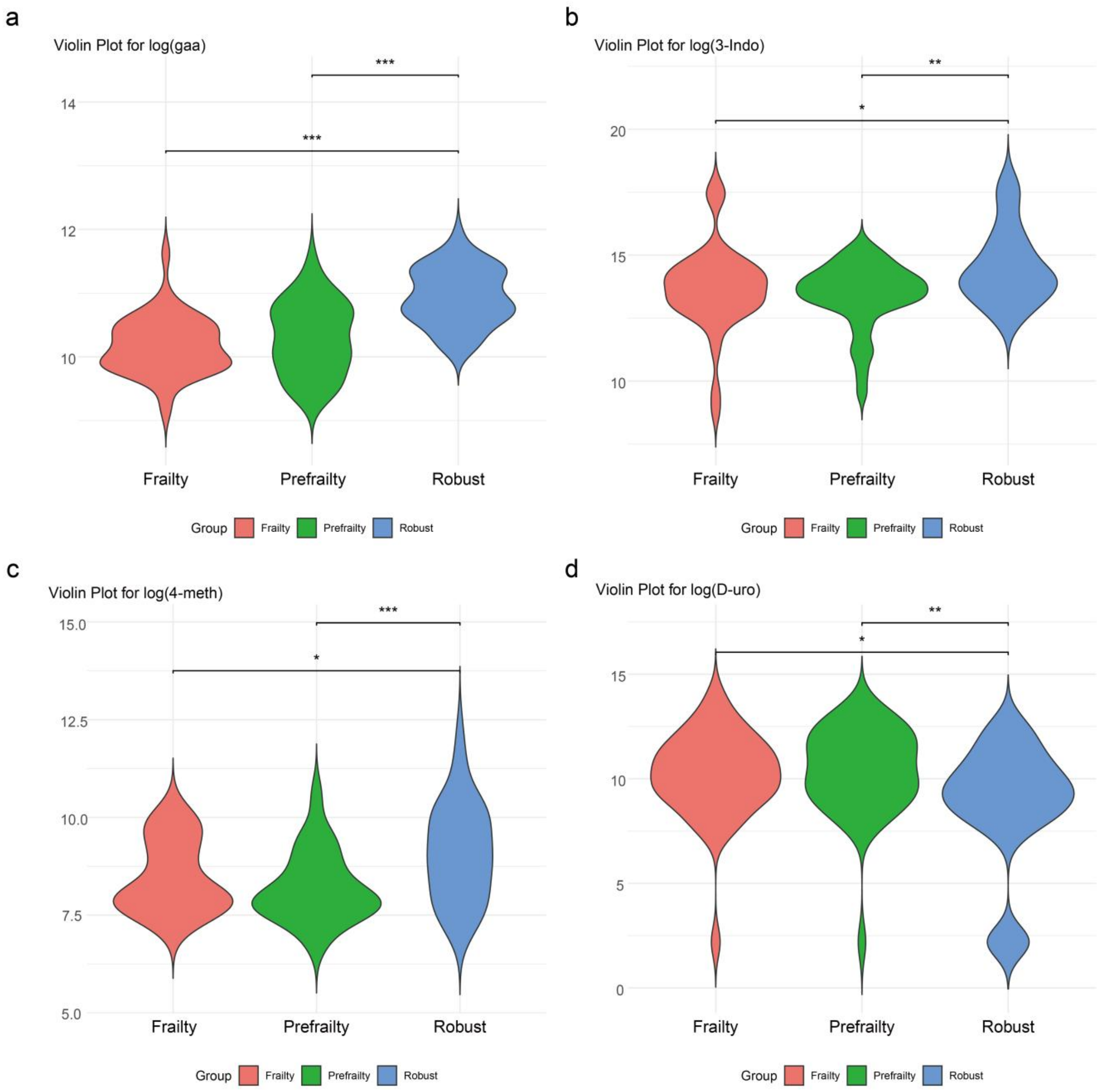

eFigure 4 Violin plots for the log-transformed four differentiated metabolites of frailty

Note: a: guanidinoacetic acid (GAA), b: 3-Indolepropionic acid, c: 4-Methoxyphenylacetic acid, d: D-urobilinogen.

****P*<0.001, ***P*<0.01, **P*<0.05 (Kruskal-Wallis test).

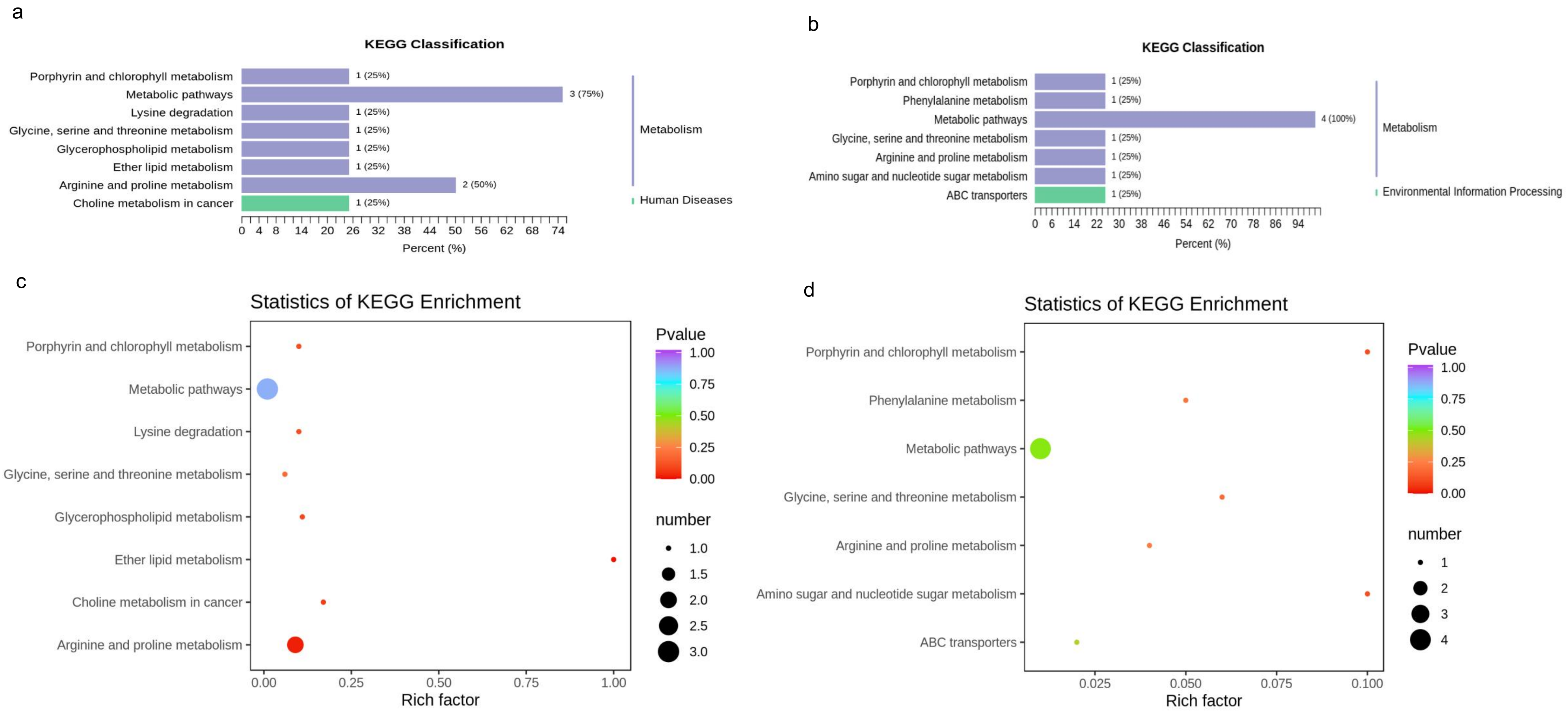

eFigure 5 KEGG pathway enrichment for differential metabolites of frailty

Note: KEGG classification for differential metabolites: a (frailty vs. robust), b (prefrailty vs. robust). KEGG enrichment bubble diagram for differential metabolites: c (frailty vs. robust), d (prefrailty vs. robust) .

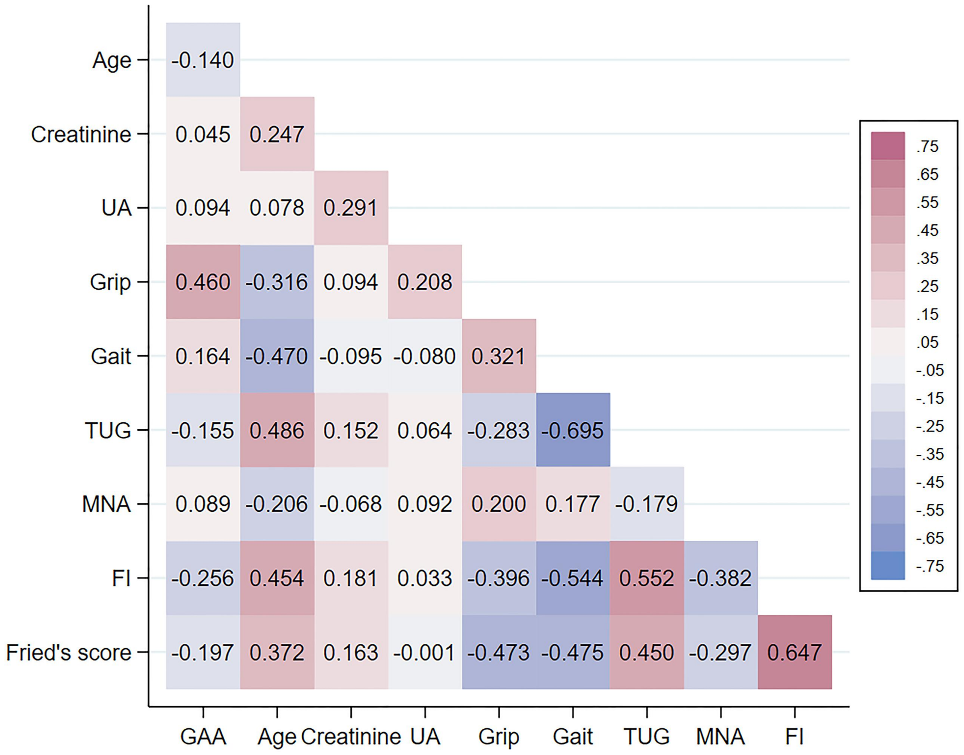

eFigure 6 Heatmap of frailty and its related factors with GAA levels

Note: UA: uric acid, Grip: grip strength, Gait: gait speed, TUG: timed up and go test, MNA: mini-nutritional assessment-short form, FI: frailty index. *P*<0.001 or *P*=0.006.

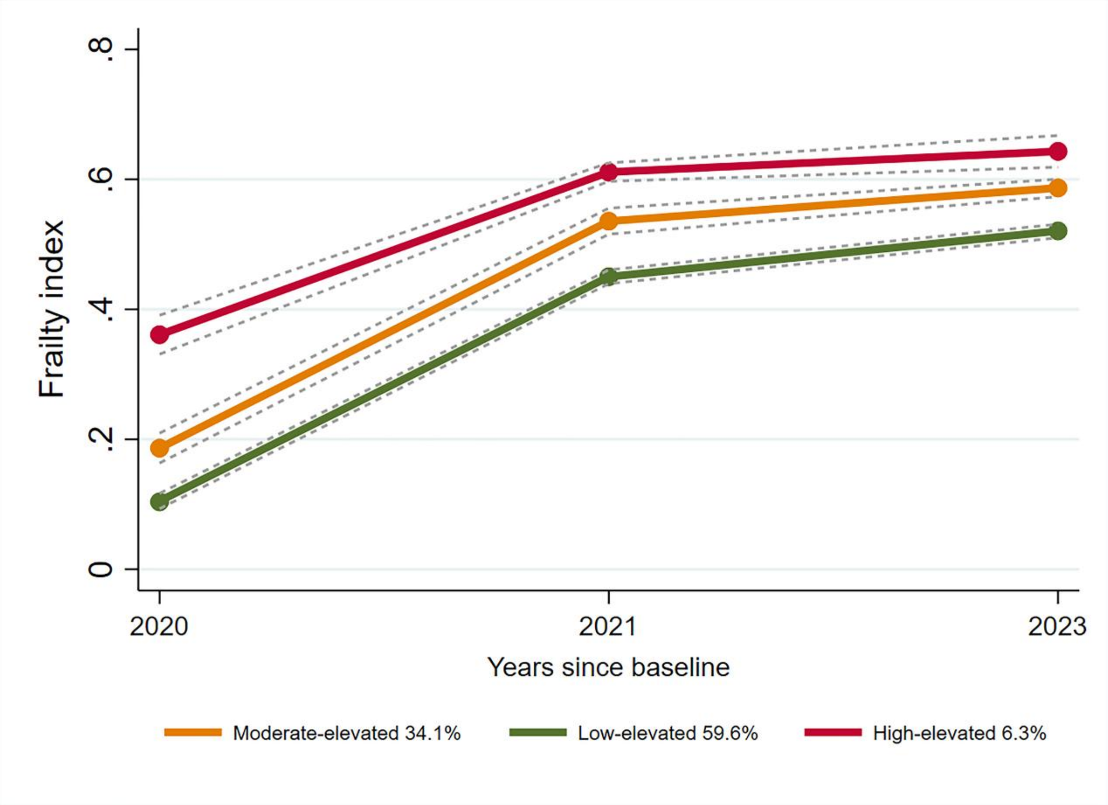

eFigure 7 The trajectory of the Frailty Index over a three-year follow-up (2020–2023)

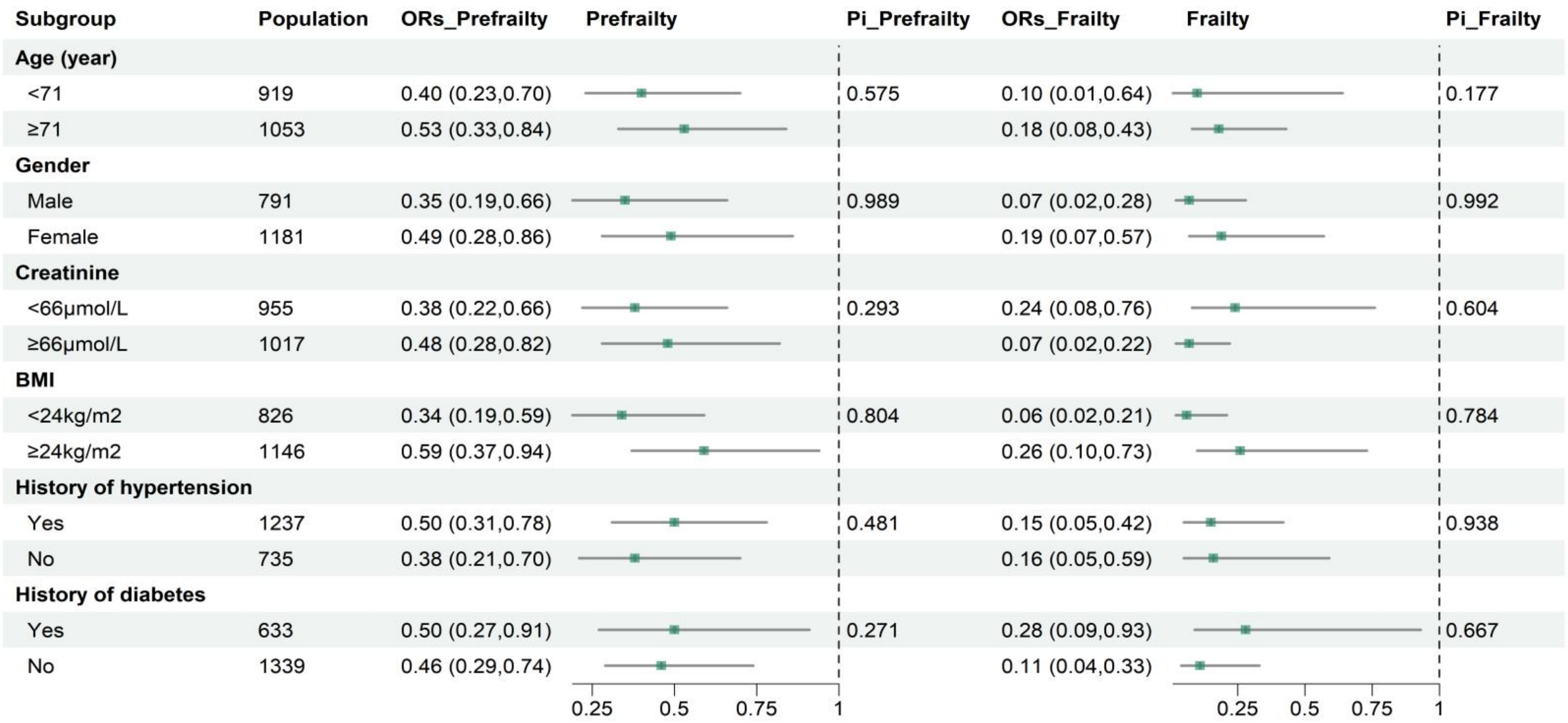

eFigure 8 Subgroup analysis of the association between the highest quartile of guanidinoacetic acid (GAA) and prefrailty and frailty (N=1972)

Note: ORs: odds ratios, Pi: *P* value for interaction.
